# Titanosaur Osteoderms from the Upper Cretaceous of Lo Hueco (Spain) and Their Implications on the Armor of Laurasian Titanosaurs

**DOI:** 10.1371/journal.pone.0102488

**Published:** 2014-08-13

**Authors:** Daniel Vidal, Francisco Ortega, José Luis Sanz

**Affiliations:** 1 Unidad de Paleontología, Universidad Autónoma de Madrid, Cantoblanco, Madrid, Spain; 2 Grupo de Biología Evolutiva, Universidad Nacional de Educación a Distancia, Madrid, Madrid, Spain; University of Pennsylvania, United States of America

## Abstract

Titanosaurs are the only sauropod dinosaurs known to bear a dermal armor. Their osteoderms are relatively rare finds, with few more than a hundred specimens recovered worldwide. Also, little is known about their intra-individual, intra-specific or inter-specific variability. The macrovertebrate site of Lo Hueco (Upper Cretaceous; Cuenca, Spain) has yielded several complete specimens of osteoderms, some associated with fairly articulated specimens. They are all variations of the morphotype known as bulb and root. The presence of only this morphotype in Europe, which is considered as the primitive condition among titanosaurs, seems to indicate that the known Upper Cretaceous Laurasian titanosaurs only bore these referred bulb and root osteoderms. An eliptic Fourier analysis on the outline of complete specimens from this morphotype reveals: i) that they truly are part of a morphological cline; and ii) the existence of a consistent correlation between the outline and the morphology of the bulb. Such variation along a cline is more consistent with intra-individual rather than inter-specific variation. The osteoderms associated with a single titanosaur individual from Lo Hueco reinforce this hypothesis.

## Introduction

Titanosaurs were a group of sauropod dinosaurs with worldwide distribution that radiated during the Cretaceous [1]. They were the only sauropods that truly developed a dermal armor [2].

The first evidence of an armored titanosaur was an osteoderm from the Upper Cretaceous found in Madagascar [3]. Piveteau reinterpreted the evidence as an ornithischian osteoderm [4], and the idea of armored titanosaurs was abandoned for many years. However, Bonaparte & Powell reported the first unambiguous evidence of an armored titanosaur [5] and, since then, titanosaur osteoderms have been found in South America, Africa, Madagascar, India, Pakistan and Europe [2], [6]–[10]. Despite all the evidence, most sites yield at most a handful of specimens and not in anatomical connection to the other skeletal remains [2], even those associated with fairly complete skeletons [11].

The Upper Cretaceous site of Lo Hueco (Cuenca, Spain) has yielded several specimens of titanosaurs, in various degrees of articulation and completeness, being the most common tetrapods in the assemblage [12]. Preliminary comparisons indicate the presence of, at least, two titanosaur species in Lo Hueco based upon two distinct types of cranial morphologies, appendicular bones and teeth. Up to now, there is no clear relationship between the remains, and therefore it is not possible to correlate the distinct types of teeth with the postcranial sets of bones and the isolated braincases at the moment. Some cranial elements present affinities with the French titanosaur *Ampelosaurus* [13] and others with the Indian *Jainosaurus* [14]. The recognized tooth morphotypes can be respectively correlated with the robust spatulated teeth of the titanosaur from Massecaps [15] and the teeth of *Atsinganosaurus* [16].

The sets of axial and appendicular skeletons available fall into two morphotypes containing autapomorphies respect to the three titanosaurs currently known in the Ibero-Armorican domain (*Ampelosaurus*, *Atsinganosaurus* and, particularly the Iberian *Lirainosaurus*). Although the analysis is still in progress, it is considered preliminarily that the remains of the two titanosaurs from Lo Hueco correspond to new taxa to be described. Moreover, it is not possible to establish the minimum number of individuals attributable to each taxa. An approximation based on the more than seventy femora collected states that the minimum number of titanosaur individuals represented in the association is above forty. However, the number of partially articulated individuals is twenty. Among them, one titanosaur specimen was found in close association with two osteoderms, although up to eighteen isolated osteoderms attributable to titanosaur sauropods have also been recovered in the site.

In this paper we describe the titanosaur osteoderms from Lo Hueco, comparing them to the worlwide known osteoderm record attributed to titanosaurs and discuss the previously published classification systems. We also evaluate their possible body arrangement based on locality data, an eliptic Fourier analysis on the outline of the osteoderms and a cluster analysis.

## Materials

The eighteen osteoderms retrieved are housed at Museo de las Ciencias de Castilla-La Mancha, Cuenca (Spain). Their specimen numbers are listed in Table 1. All the osteoderms from Lo Hueco are identified as titanosaurian on the basis of the absence of an attachment area for tendons [2] and the presence of internal keel/s, a feature that is postulated as a synapomorphic trait of armored titanosaurs.

**Table 1:** Osteoderms from Lo Hueco.

Specimen	Length	Width	Bulb type
HUE 00561	241	170	Flat
HUE 00590	292	180	Flat
HUE 00913 (HUE-EC-11)	250	150	Flat
HUE 00950 (HUE-EC-11)	520	155	Concave
HUE 01330	505	144	Concave
HUE 02000	390	105	Convex
HUE 02029	230*	175	Flat
HUE 02326	270	117	Convex
HUE 02900 (HUE 02309)	210*	190	Flat
HUE 04505	295	150	Flat
HUE 08393	270	140	Convex
HUE 08738	90*	90*	Convex

The twelve analyzed osteoderms from Lo Hueco, with main measures in millimeters. The specimens associated with the articulated titanosaur individuals HUE-EC-11 or HUE-02309 are indicated. Measurements with an *asterisk indicate a broken axis of the specimen measured.

### Terminology and classification

**Figure 1:**
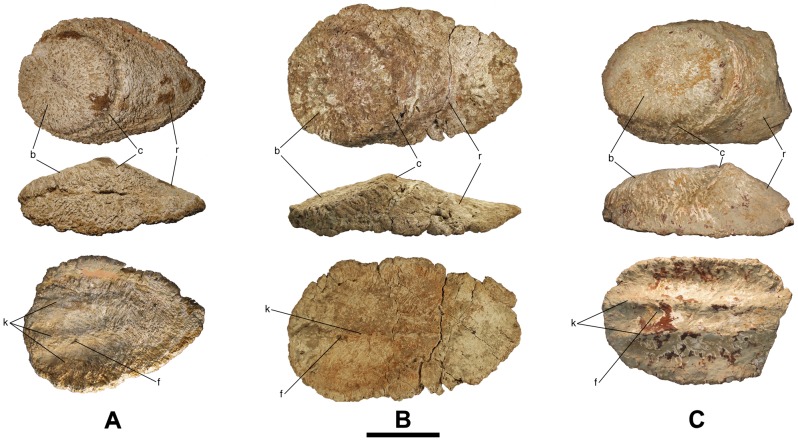
Bulb and root osteoderms with a flat bulb from Lo Hueco I. A - HUE-00561 in external (top), lateral (middle) and internal (bottom) views. B - HUE-00590 in external (top), lateral (middle) and internal (bottom) views. C - HUE-02029 in external (top), lateral (middle) and internal (bottom) views. b = bulb; c = cingulum; f = foramina; k = keel; r = root. Scale = 10 cm.

**Figure 2:**
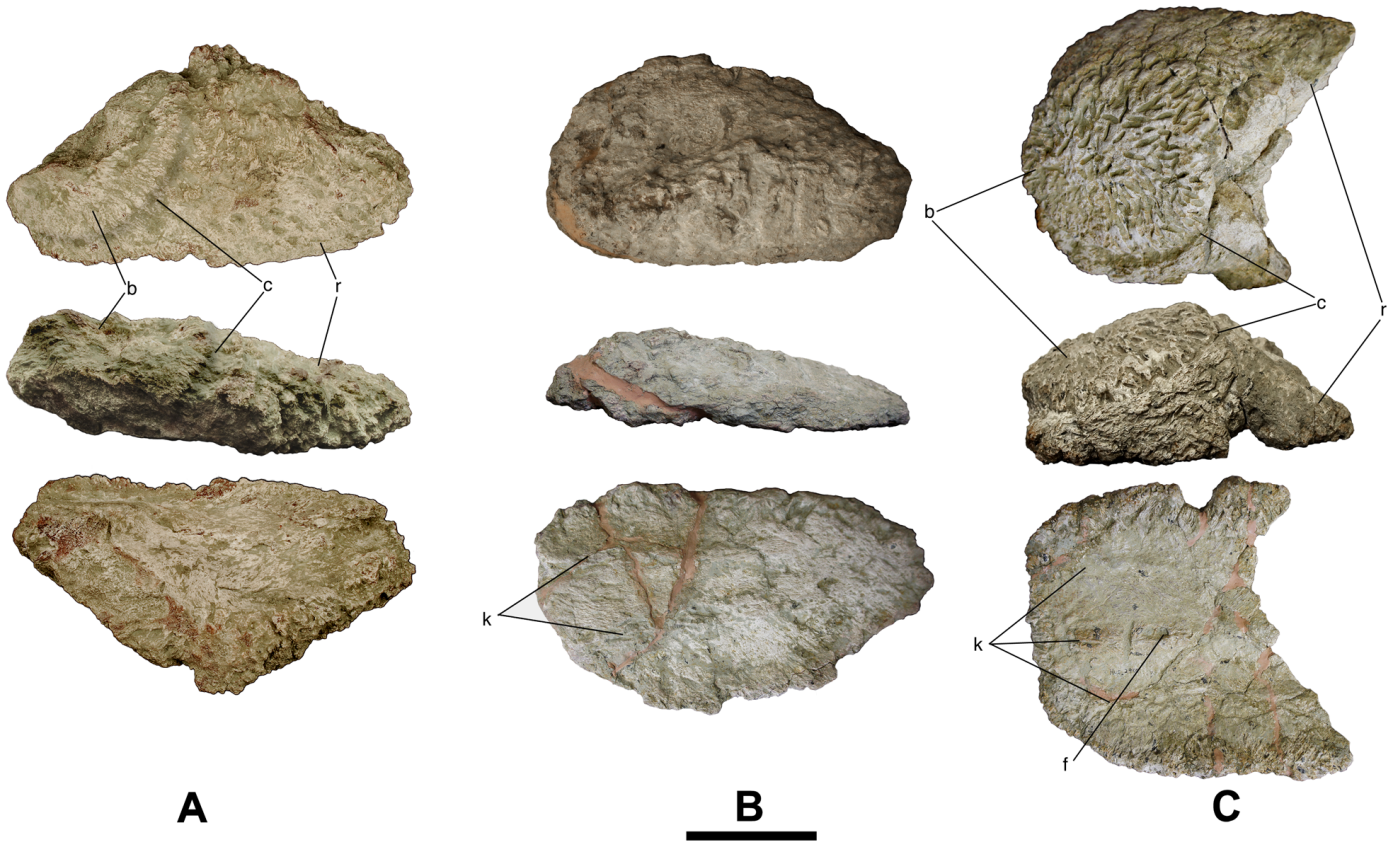
Bulb and root osteoderms with a flat bulb from Lo Hueco II. A - HUE-04505 in external (top), lateral (middle) and internal (bottom) views. B - HUE-00913 in external (top), lateral (middle) and internal (bottom) views. C - HUE-02900 in external (top), lateral (middle) and internal (bottom) views. Scale = 10 cm.

**Figure 3:**
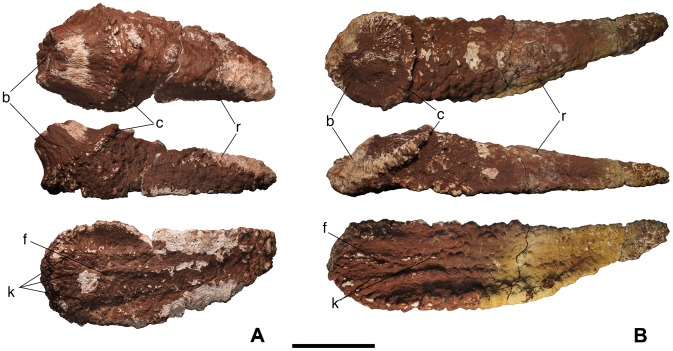
Bulb and root osteoderms with a convex bulb from Lo Hueco. A - HUE-02326 in external (top), lateral (middle) and internal (bottom) views. B - HUE-02000 in external (top), lateral (middle) and internal (bottom) views. Scale = 10 cm.

**Figure 4:**
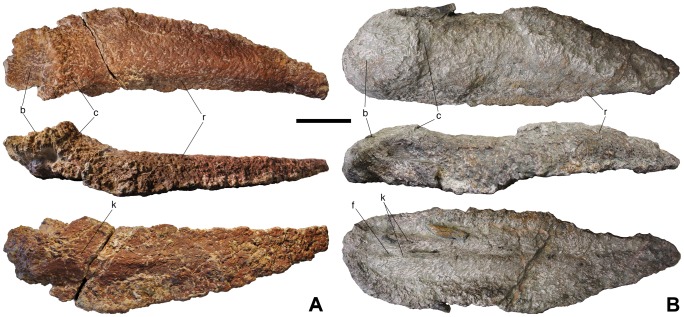
Bulb and root osteoderms with a concave bulb from Lo Hueco. A - HUE-01330 in external (top), lateral (middle) and internal (bottom) views. B - HUE-00950 in external (top), lateral (middle) and internal (bottom) views. Scale = 10 cm.

The term osteoderm is used for dermal bones bigger than 1 cm and ossicles for dermal bones smaller than 1 cm, as in Arbour et al. [17]. We use the anatomical terms of internal and external according to D'Emic et al. [2], for referring to the surfaces of the osteoderm towards and away from the body, respectively. The term lateral refers to the sideviews. The terms bulb and root refer to two distinct regions of the osteoderm in external and lateral views, well delimited by a cingulum (Figs. 1–4).

As for the classification, we use that of D'Emic et al. [2], with slight variations (see discussion). All the institutional abbreviations from previously published specimens of all morphotypes discussed here are listed on Table S2.

### Description

Every osteoderm retrieved from Lo Hueco belongs to the bulb and root morphotype [2]: they have an amygdaloid outline, a convex external side and a concavo-convex internal side, with two distinct, well differentiated regions in lateral and external views: the bulb and the root (Figs. 1–4). In external or internal views, most specimens have a straight side whereas the other is more curved. The bulb is displaced toward the straightest side, giving the specimens a peculiar asymmetry. Some specimens are almost symmetrical (i.e. HUE-00561, Fig. 1 A), with both sides straight and without the bulb displaced.

**Figure 5:**
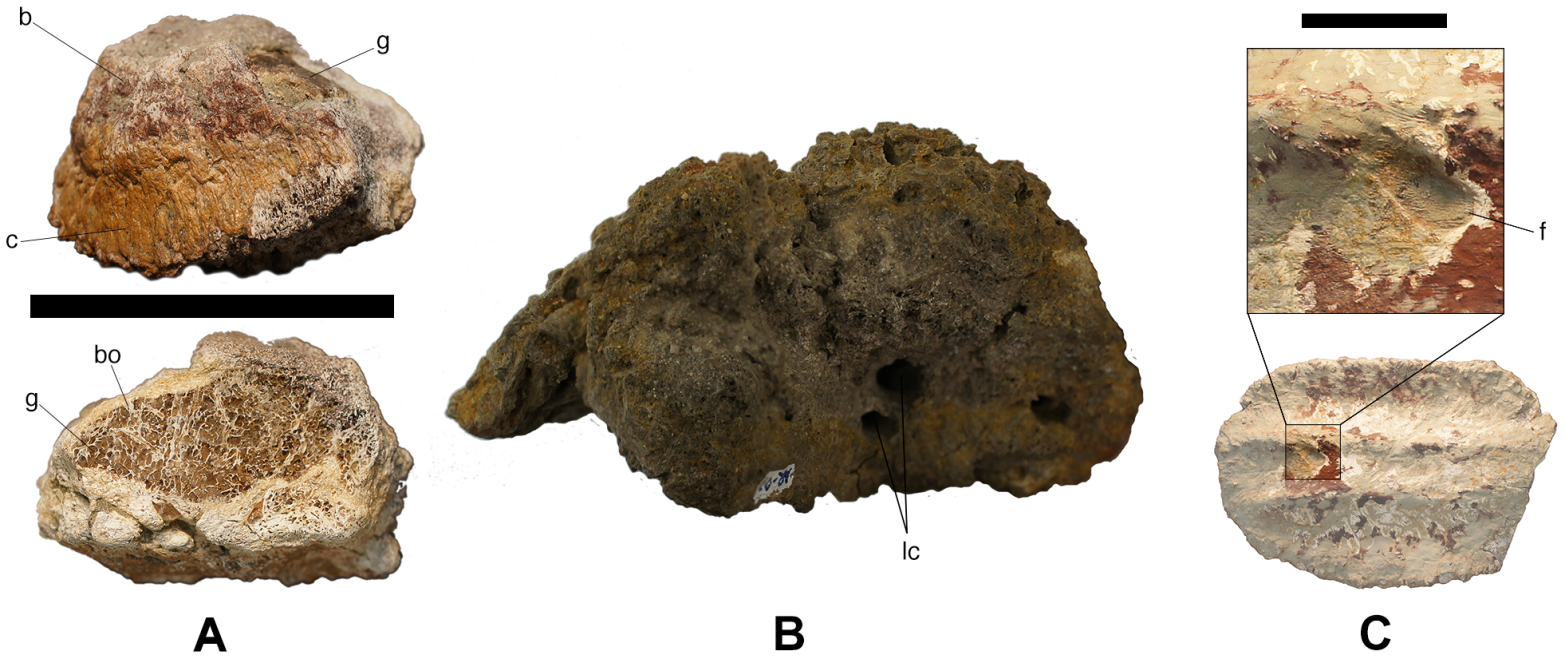
Details of titanosaur bulb and root osteoderms. A - Top: HUE-08738 in lateral view. Bottom: Section of HUE-08738, revealing a large cavity filled with gypsum. B - Section of UPUAM-13952, revealing longitudinal channels. C - The largest foramen found in the internal surface of bulb and root osteoderms, right under the bulb (filled with sediment). bo = bone; f = foramen; g = gypsum deposit; lc = longitudinal canals. Scale = 10 cm (A and B), 1 cm (C).

All the available fragmentary specimens can be identified as parts of bulbs, roots or both. Their measurements are summarized in Table 1. Their internal anatomy can be preliminarily explored in broken specimens. These specimens reveal that some osteoderms had large cavities inside while some were compact with or without longitudinal canals (Fig. 5 A–B). However, a more detailed analysis using radiographies, CAT scans or histological slices remain to be performed.

In internal view, they range from flat to slightly concave and display a woven texture: a pattern of intersecting, criss-crossed bone fibers, common in archosaur osteoderms [2]. All of them present up to three internal keels, which are slightly displaced toward the straightest side of the osteoderm in the asymmetric specimens. They present nutrient foramina of different sizes as well, with the largest, up to 2 cm in diameter, always located under the bulb (Fig. 5 C).

In external view, the bulb is a sub-circular region, well delimited by a cingulum. It has a regular pattern of radiating fibers or pits. The root limits with the bulb, reaching its maximum width and tapering away from the bulb. Its length is variable, ranging from twice to up to five times the length of the bulb. Its texture is more irregular, with nodules, fibers and grooves.

In lateral view, the bulb presents different morphologies [18]: spines (convex or conical), flat or concave. The root reaches its maximum thickness at the cingulum, tapering away from the bulb. For the present study we have decided upon using the terms convex, flat and concave to describe the surface of the bulb in lateral view. The specimens from Lo Hueco show all of the aforementioned bulb morphologies:

#### Flat bulb

Externally, their outline is amygdaloid and quite rounded, symmetrical in HUE-00561 (Fig. 1A), slightly asymmetric in HUE-02029 (Fig. 1C) and quite asymmetric in HUE-00590 (Fig. 1D). HUE-00913, 02900 and 04505 (Fig. 2) are broken, but the former two appear to have been asymmetric as one side is quite straight while the other is curved, whereas HUE-04505 is very symmetric. In lateral view, HUE-00561, 00590, 00913 and 04505 are flattened whereas HUE-02029 and 02900 are quite thicker. All the specimens present a pattern of radiating foramina but not fibers in the bulb. In the root, HUE-00561, 00590, 02029 and 02900 present a pattern of intersecting fibers and channels but not nodules while HUE-00913 and 04505 present fibers, nodules and grooves. HUE-00561 and 00590 present three shallow keels in internal view, displaced to the straightest side on HUE-00590. HUE-00913 has a shallow bilobate keel. HUE-02029 has a thick, bilobate keel, being its apex the most internal point of the osteoderm. The internal surface of HUE-00913 and 02900 cannot be observed due they are broken.

HUE-02029 is broken in the root region, revealing its internal structure, which is quite compact, without any duct or large openings. The bone is spongy with the microcavities filled with gypsum.

#### Convex bulb

Externally their outline is amygdaloid and ranges from slightly to quite elongated, all of them asymmetric, having a straight and a curved side. The bulb presents a radial ornamentation of fibers in HUE-02326 (Fig. 3. A) and 08738, and foramina in HUE-02000 (Fig. 3 B). In lateral view, the height of the bulb is greater than that of the root in HUE-02326 and 08738, and about the same height in HUE-02000. Internally, HUE-02000 and 02326 present 3 keels. HUE-08738 is broken and has not preserved most of the root or the internal side. The fracture reveals a large gypsum deposit, embedded in the large openings in the spongy bone of the osteoderm (Fig. 5 A). Such a cavity seems similar to that reported by Curry-Rogers et al. in an osteoderm with a flat bulb [8]. HUE-08393 was collected one year after the main dig and thus was affected by meteorization. Despite its surface being too eroded, the general morphology of the bulb can be determined as convex.

#### Concave bulb

The most remarkable osteoderms retrieved at Lo Hueco are the very elongated HUE-00950 and HUE-01330 (Fig. 4). The surface of the bulb is concave, totally in HUE-01330 and only in the center of the bulb in HUE-00950, with a radial ornamentation of foramina. The cingulum is well developed in both osteoderms and the root is about five times longer than the bulb. Internally, HUE-01330 presents a thick, wide keel whereas HUE-00950 presents a thick, bilobate keel. Both are broken, with their sections revealing compact bone without any large openings or ducts.

## Methods

### Morphological analysis methods

Morphological analysis of the specimens was carried out using an elliptic Fourier analysis (EFA). Given the high disparity observed in the outlines of the specimens (from rounded to elongated osteoderms), the aim of the analysis was to find out whether bulb and root osteoderms could be splitted in distinct morphotypes or not. In order to limit the amount of speculation in reconstructing the outlines, only the eighteen published bulb and root osteoderms that were complete enough were included in the analysis (Table 2).

**Table 2:** Bulb and Root osteoderms used in the elliptic Fourier analysis.

Site(s)	Specimens	Age	Source
Lo Hueco (Spain)	HUE 00561	Ca-Ma	[29]
	HUE 00590		
	HUE 00913		
	HUE 00950		
	HUE 01330		
	HUE 02000		
	HUE 02326		
Aude (France)	MDE-C3-136	Ma	[18]
	MDE-C3-192		
	MDE-C3-325		
Hateg (Romania)	FGGUB R.1410	Ma	[25]
Mwakasyunguti (Malawi)	MAL-204	Ap	[31]
Malagasy (Madagascar)	FMNH PR 2342	Ma	[8]
Cinco Saltos (Argentina)	Placa Dérmica Gigante	Ca	[24]
Cruzy (France)	CRU 1, 2 and 3	Ma	Unpublished
Vitrolles (France)	CM 536		Unpublished

To perform the analysis, the data from the outline of every osteoderm was converted to coordinates in a Cartesian System, a hundred per each specimen, using tpsDIG2 freeware (Coordinates available on Table S1).

Following the recommendations of Hammer & Haper [19], every osteoderm was given the same orientation, with the widest portion (the bulb) always on the left side and the straightest side parallel to a straight line. In order to avoid the influence of the bulb morphology, images in internal view were used whenever possible.

The coordinates were analyzed in PAST [20], obtaining the first 30 harmonics and the variable “size” of each osteoderm. A principal components analysis (PCA) was performed in order to further reduce the number of variables from the EFA and study the variation in a XY graph.

HUE-00913 (Fig. 2B) and HUE-00950 (Fig. 4B) are both associated to the same titanosaur specimen (HUE-EC-11), and might give an insight on how much of the morphological variation of the osteoderms was present in the same individual. However, HUE-00913 is not complete and cannot be included in the EFA. In order to study those osteoderms which are not complete and contrast the EFA as well, a cluster analysis from qualitative characters (Table 3) from the osteoderms of Lo Hueco was carried out in PAST, with the paired group algorithm and the Jaccard index for similarity measure.

**Table 3:** Characters x osteoderms matrix used for the cluster analysis.

	1	2	3	4	5	6	7	8
HUE 00561	0	0	0	0	1	0	1	0
HUE 00590	0	0	0	1	0	0	3	0
HUE 00913	?	?	?	1	0	0	2	?
HUE 00950	2	0	1	1	1	0	2	1
HUE 01330	2	0	1	1	1	1	1	1
HUE 02000	1	0	1	1	1	1	3	1
HUE 02029	0	0	?	1	1	0	2	0
HUE 02326	1	1	1	1	1	1	3	1
HUE 08393	1	1	?	1	?	?	?	1
HUE 02900	0	1	?	1	0	0	2	0

The qualitative characters for the cluster analysis were: 1) Intracingular morphology of the bulb: flat (0), convex (1) concave (2); 2) Intracingular radial ornamentation of the bulb: pitting (0), fibers (1); 3) General contour in internal view: widen (0), elongated (1); 4) Symmetry: bilateral (0), asymmetric (1); 5) Root dorsoventrally flattened (0), not flattened (1); 6) External ornamentation of the root: rugose, interwoven fibers without nodules (0), perlated, with nodules (1); 7) Number of keels in the internal side: one (1), two (2), three (3); 8) Type of cingulum: excavated border (0), platform (1).

## Results

**Figure 6:**
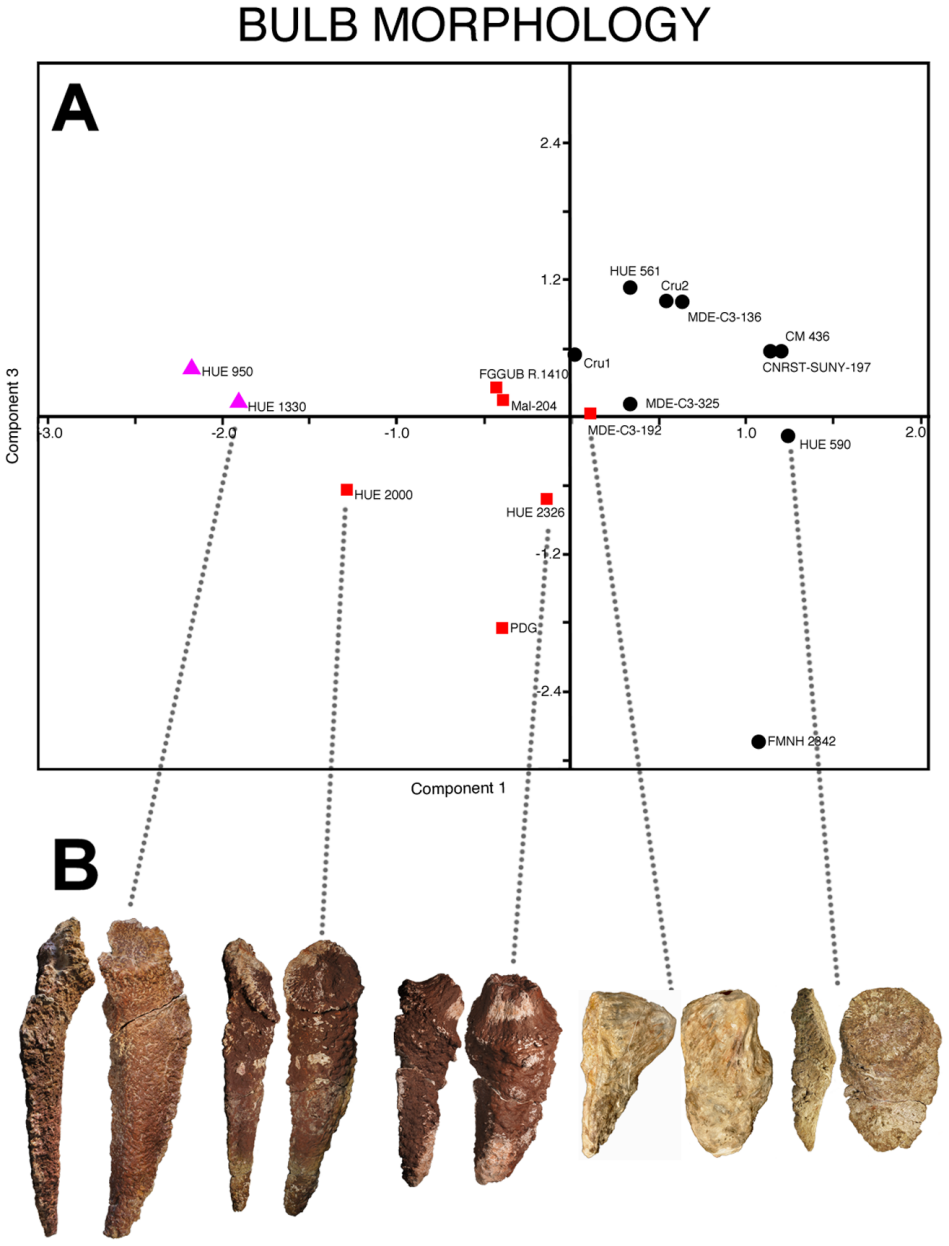
Morphological cline of the bulb and root osteoderms. A - Scatter plot with the first and third principal components obtained from the principal components analysis, using the data of an elliptic Fourier analysis on the outline of the osteoderms, with the morphology of the bulb highlighted: triangle = concave bulb; square = convex bulb; circle = flat bulb. B - Bulb and root osteoderm specimens located in the scatter plot. From left to right, HUE-01330, HUE-02000, HUE-02326, MDE-C3-192 and HUE-00590.

The EFA output shows that, upon their silhouette in internal view, bulb and root osteoderms are evenly distributed in a morphological cline (Fig. 6). The first principal component (PC1) explains more than 99% of the variance, with the rest of components explaining less than 1%. Considering that the variable “size” contributes the most to PC1 and that all the osteoderms were scaled to the same length we interpreted it as the relative width of the osteoderms, and thus as a general measure of their shape: those osteoderms with negative PC1 scores are more elongated and those with more positive scores are more rounded. The third principal component (PC3) was found to correlate well with the symmetry of the specimens in the scatter plot, and was thus interpreted as a symmetry parameter: osteoderms with positive scores are more symmetric than those with more negative scores.

Among this morphological cline, three different groups of osteoderms can be identified if the morphology of the bulb is highlighted in the scatter plot (Fig. 6A). Osteoderms with a flat bulb are more rounded, with lengths not much greater than their widths; osteoderms with a convex bulb have greater lengths than widths and those with a concave bulb have enormous roots, up to five times the length of the bulb. There are “transitional” osteoderms between these groups, with slightly convex bulbs yet quite elongated roots (HUE-02000, Fig. 6A).

**Figure 7:**
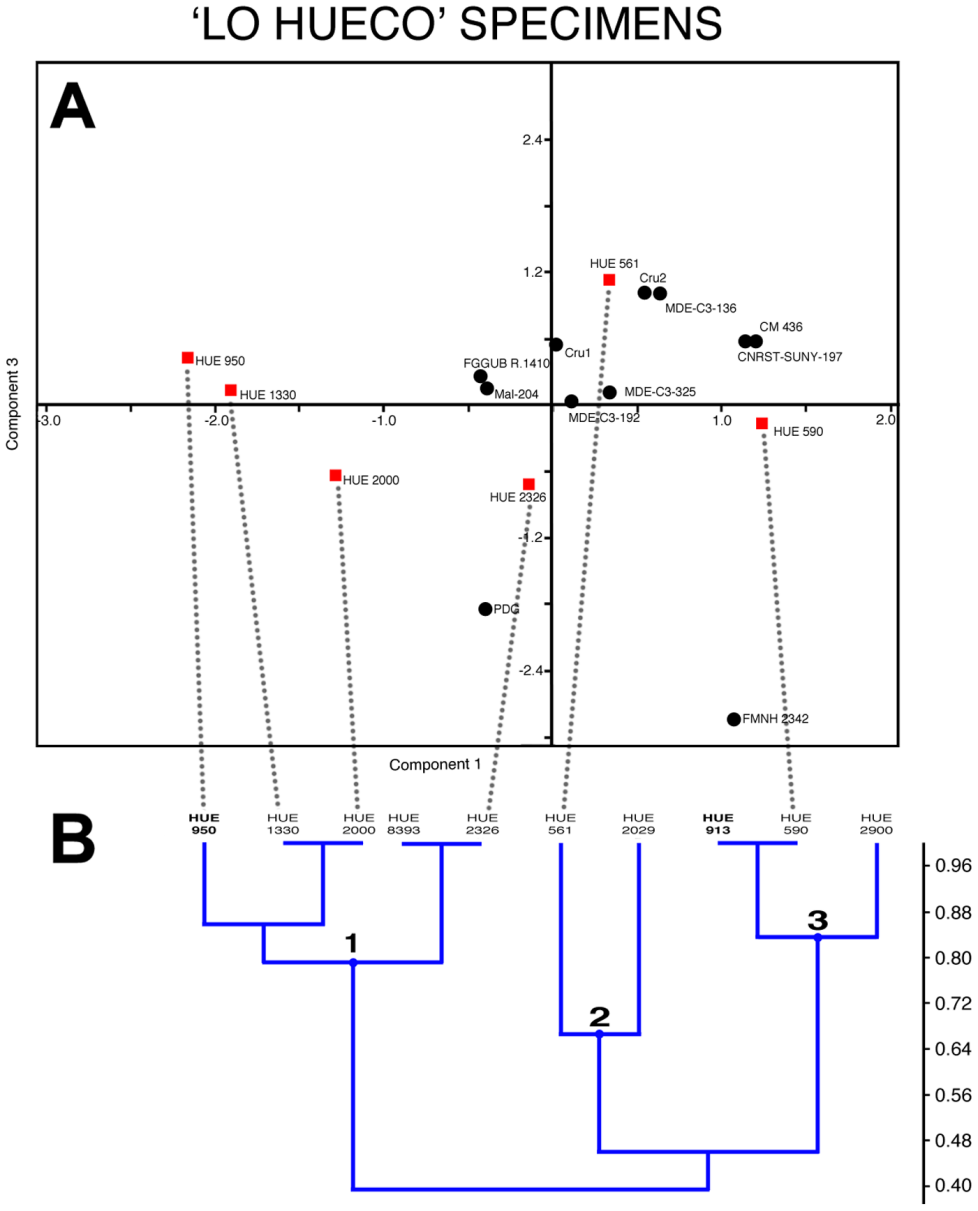
Comparison between the elliptic Fourier and cluster analysis of the bulb and root osteoderms. A - Scatter plot with the first and third principal components obtained from the principal components analysis, using the data of an elliptic Fourier analysis on the outline of the osteoderms, with the specimens from Lo Hueco highlighted (as red squares). B - Dendrogram obtained from the cluster analysis on those specimens of Lo Hueco complete enough to have >50% of the characters (Table 3). Highlighted in bold are the osteoderms associated with the titanosaur individual HUE-EC-11.

The osteoderms from Lo Hueco are scattered throughout the graph, thus being representative of the worldwide fossil record of titanosaur bulb and root osteoderms (Fig. 7).

The cluster analysis separates the osteoderms into 3 main clusters: one includes elongated osteoderms with either concave or convex bulbs (Fig. 7.1), another includes thick osteoderms with flat bulbs (Fig. 7.2), and the third cluster includes flattened osteoderms with flat bulbs (Fig. 7.3). The last two clusters are part of a more inclusive but not so well supported cluster, which would include all the osteoderms with a flat bulb. If compared to the EFA, the distribution and clustering is quite similar (Fig. 7), and thus HUE-00913 and HUE-00950 appear to have displayed different morphologies, suggesting that bulb and root osteoderms of different outlines and bulb morphologies were associated with the same individual.

## Discussion

### Classification and identification

**Figure 8:**
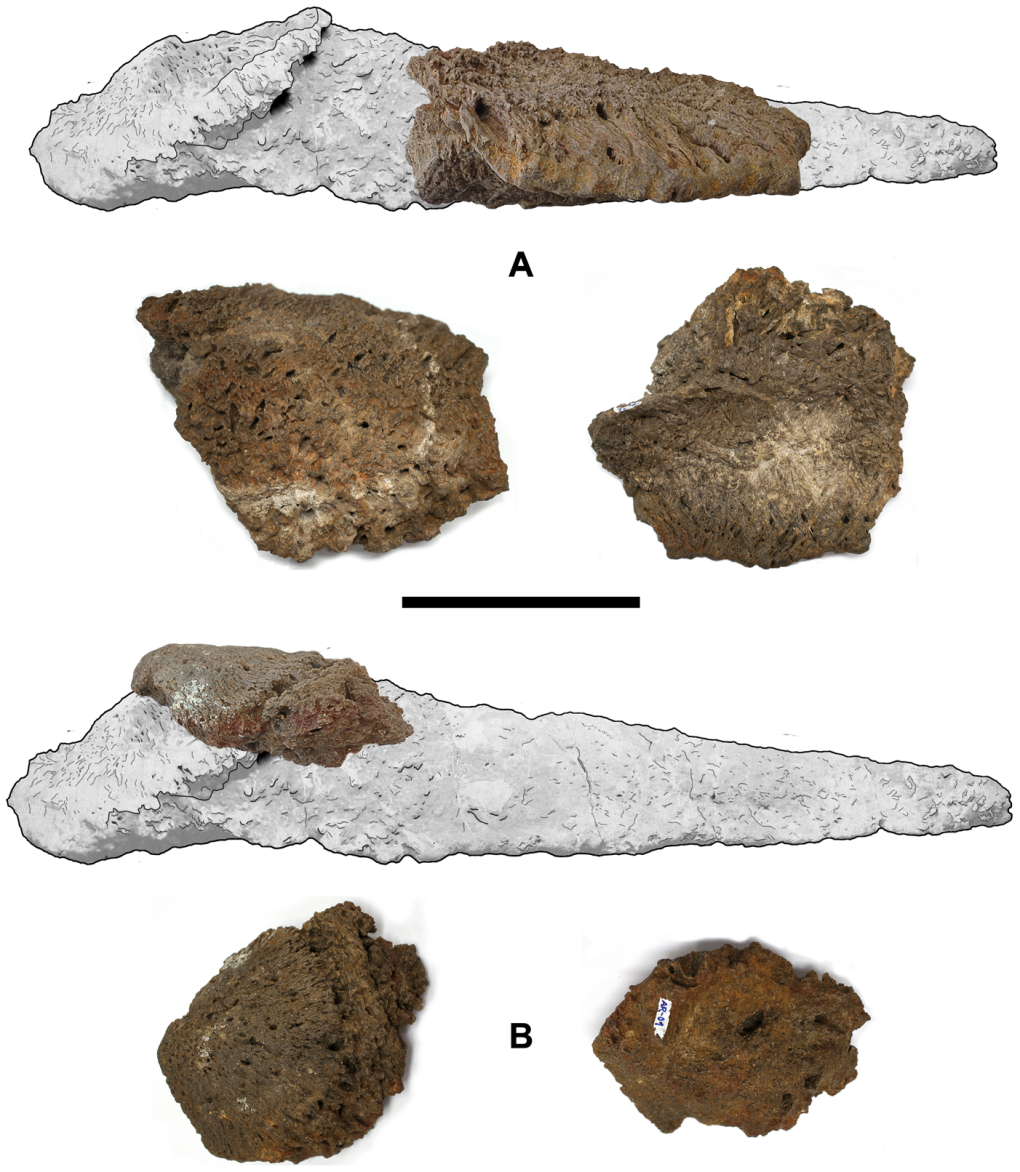
Fragmentary specimens from the Armuña locality reinterpreted as bulb and root osteoderms. A - UPUAM-13952 in lateral, interpreted as part of a root (top), external (bottom, left) and internal (bottom, right) views. B - UPUAM-13951 in lateral, interpreted as a bulb and a cingulum (top), external (bottom, left) and internal (bottom, right) views. Scale = 10 cm.

The most comprehensive and complete classification of osteoderms attributable to titanosaurs was proposed by D'Emic et al. [2]. This classification established four different morphotypes based on the published fossil record of titanosaur osteoderms: ellipsoid, keeled, cylindrical and mosaic. Curry-Rogers et al. proposed that many osteoderms identified as part of the mosaic morphotype might have been fragments of larger osteoderms [8]. As broken specimens are always susceptible of reinterpretation (Fig. 8), in this paper we suggest a variation in D'Emic et al. system, based upon complete specimens only. Moreover, as noted by Arbour et al. [17], there should be a distinction between osteoderms (dermal bones greater than 1 cm) and ossicles (dermal bones smaller than 1 cm), as they have been proven to be different both in morphology [2] and histology [21]. Among the osteoderms, we propose the following classification:

#### Morphotype 1 - bulb and root

Osteoderms with amygdaloid outline and the general appearance of a barrel vault, with two well differentiated regions in lateral and external views: the bulb and the root, delimited by a cingulum. The bulb has a surface texture made of a radial pattern of fibers or foramina while the root presents a more irregular pattern of ducts and nodules. Their base (internal side) is slightly concave, with up to three ridges or keels, which range from slight to quite thick.

D'Emic et al. stated that some ellipsoid osteoderms are made up only of the “bulb” or the “root” regions [2]. However, some of these specimens seem to be broken, and could be interpreted as parts of a complete bulb and root osteoderm. This would be the case of the Armuña (Segovia, Spain) specimens UPUAM-13951 and UPUAM-13952 (Fig. 8, see [28], referred respectively as AR-01 and AR-02 in [2]), FSL 92827, UA 8675 or MN-5013V (see [2]).

**Figure 9:**
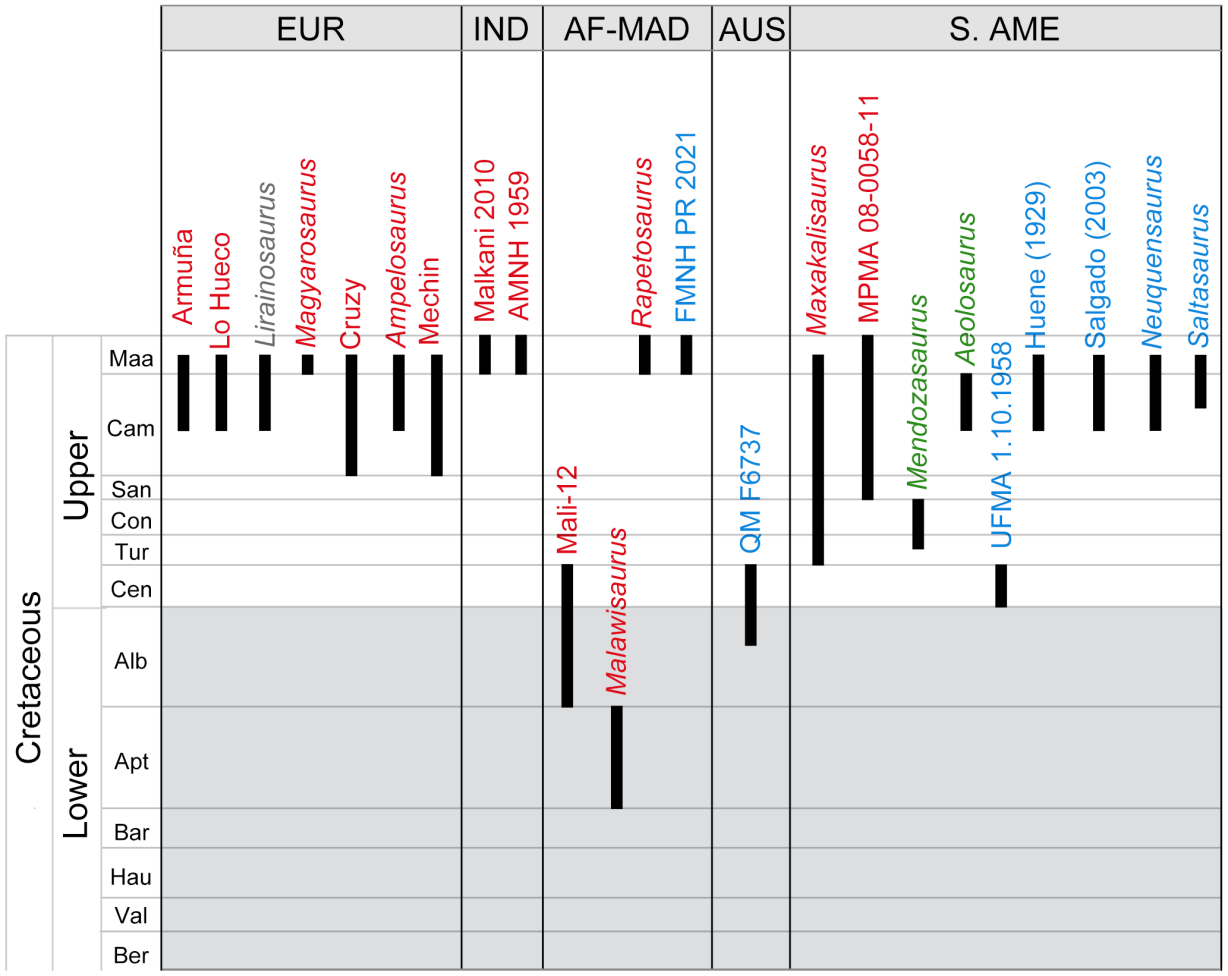
Spatiotemporal calibration of the titanosaur osteoderm fossil record. The morphotypes are highlighted in colors. Red: bulb and root. Blue: scutes. Green: Controversial elements. Gray: The osteoderms of *Lirainosaurus*, too fragmentary to interpret.

This morphotype would include most of the ellipsoid osteoderms *sensu* D'Emic et al., MPMA 08-0058-11 [9] and MSM-84-7 and MSM-85-4 [6]. FSL 92827 and UA 8675, both cylindrical osteoderms *sensu* D'Emic et al., are here interpreted as a bulb and a root, respectively. Bulb and root osteoderms are found in Europe, India, South America, Africa and Madagascar (Fig. 9).

#### Morphotype 2 - scutes (or keeled *sensu* D'Emic et al.)

These osteoderms present one region of sub-circular contour with a radial pattern of foramina or fibers, sometimes surrounded by a cingulum or a perlated contour. In lateral view, they might present an external keel, a convex or a flat morphology. They could be interpreted as bulbs that somehow lost the root portion. However, there is not enough evidence to support such homology.

We interpret this group of osteoderms as in D'Emic et al. However, the presence of internal keels is not unique to this morphotype, as it is present in bulb and root osteoderms from Spain, France and Romania as well. Therefore, as the term “keeled” can be misleading, we propose the term “scute” for this morphotype. Aside from all the osteoderms in D'Emic et al. [2], the more recent findings UFMA 1.10.1958 [10] and the putative QM F6737 [7] would also be scutes. They have been found in Africa, Madagascar, South America and Australia (Fig. 9).

#### Controversial elements

Some osteoderms do not seem to fit in the morphotypes proposed either here or in D'Emic et al. [2]. According to photographs and descriptions, they are elongated, compressed, with an external apex and a convex internal face. However, they do not show any distinct features from a scute, and could either be part of their variability or be a distinct morphotype on their own. More detailed study on the specimens is required to clarify this issue. These osteoderms are those associated with *Mendozasaurus* [22] and *Aeolosaurus* [23], as well as the “placa dérmica pequeña” described by Powell [24]. They have only been found in Argentina (Fig. 9).

### The nature of the Laurasian titanosaur osteoderms

Considering the wide disparity displayed by the fossil record of titanosaur osteoderms, the question whether such disparity represents inter-specific, intra-specific or intra-individual variability remains unclear. The identification of the different morphotypes within the taxa in the different localities around the world is crucial to understand their phylogenetic and biogeographical distribution.

The collection of titanosaur osteoderms so far available from Europe consists of at least a complete osteoderm from Romania [25]; three complete osteoderms from Massecaps at Cruzy, France; a complete osteoderm from Vitrolles, France; four complete osteoderms from Aude, France [18]; two fragmentary osteoderms from Laño, Spain [26], [27]; five fragmentary osteoderms from Armuña, Spain [28] and seven complete and eleven fragmentary osteoderms from Lo Hueco, Spain ([29]; this paper).

The complete known specimens from the Laurasian titanosaur record are all bulb and root osteoderms, and the fragments can all be interpreted as portions of complete bulb and roots (Fig. 8). Also, there are no osteoderms from Laurasia interpreted as a scute or dermal ossicle. This could mean that the european titanosaurs only bore bore bulb and root osteoderms, if the fossil record is taken at face value.

The simultaneous presence of both bulb and roots (FMNH PR 2342) and scutes (FMNH PR 2021) occurs only in the Maevarano Formation, Madagascar, where at least two different titanosaurs were found: *Rapetosaurus krausei*, a non saltasaurine lithostrotian [1], [30] and the Malagasy Taxon B, a saltasaurine [1]. Thus far, only saltasaurine titanosaurs have been associated with scutes, so it would be likely that both titanosaurs from the Upper Cretaceous of Madagascar were armored, and bore different types of armor, thus implying that the type of osteoderms might have taxonomic and systematic value.

The bulb and root condition appears to be primitive in Titanosauria, as the morphotype is more widespread in time and space (Fig. 9). Also, basalmost forms of Lithostrotia such as *Malawisaurus* are associated with bulb and roots [31], whereas derived forms (i.e. saltasaurines) are associated with scutes and dermal ossicles, thus possibly representing a derived condition.

### Arrangement of osteoderms on the titanosaur body

The anatomical arrangement of titanosaur osteoderms has been debated since Bonaparte & Powell first proposed a tessellated arrangement of dermal ossicles surrounding the larger plates (scutes *sensu* this paper) [5]. This hypothesis was reinforced by embryonic skin impressions of titanosaurs found at Auca Mahuevo [32]–[33], where rows and rossette arrangements of scales were found. However, it is not possible to assess the exact position of those patterns in adult titanosaurs, due to the difficulty to assign the skin impressions to a precise body region. Moreover, in extant archosaurs, osteoderms are post-natal ossifications [32] and, therefore, these impressions may or may not correspond to the adult skin pattern.

The general consensus is that titanosaurs were lightly armored forms [2], [8], [28], with several proposed arrangements, such as osteoderms restricted to the sacro-pelvic region [28] or osteoderms with flat bulbs in the dorsal and sacral regions and osteoderms with convex bulbs restricted to the scapular region [18]. Salgado suggested a sagittal row for the most symmetric osteoderms and parasagittal rows for the asymmetrical specimens [34] in the light of two evidences: i) the more asymmetric the osteoderms of *Alligator mississippiensis*, the further they are located from the median plane [35] and ii) the existence of median epidermal structures in an undescribed diplodocid sauropod [36].

We also consider the asymmetrical specimens of bulb and root osteoderms to be part of parasagittal rows because of the orientation of the big nutrient foramina located in the internal side. These nutrient foramina enter obliquely the minor axis of osteoderm (Fig. 5 C), which might evidence a vascular irrigation similar to that of extant archosaurs, with a dorsal median artery that bifurcates to the left and to the right to perfuse a whole transversal row of osteoderms, median to lateral direction [35]. The more symmetric osteoderms might belong to a median arrangement that could represent a transition to a median position.

**Figure 10:**
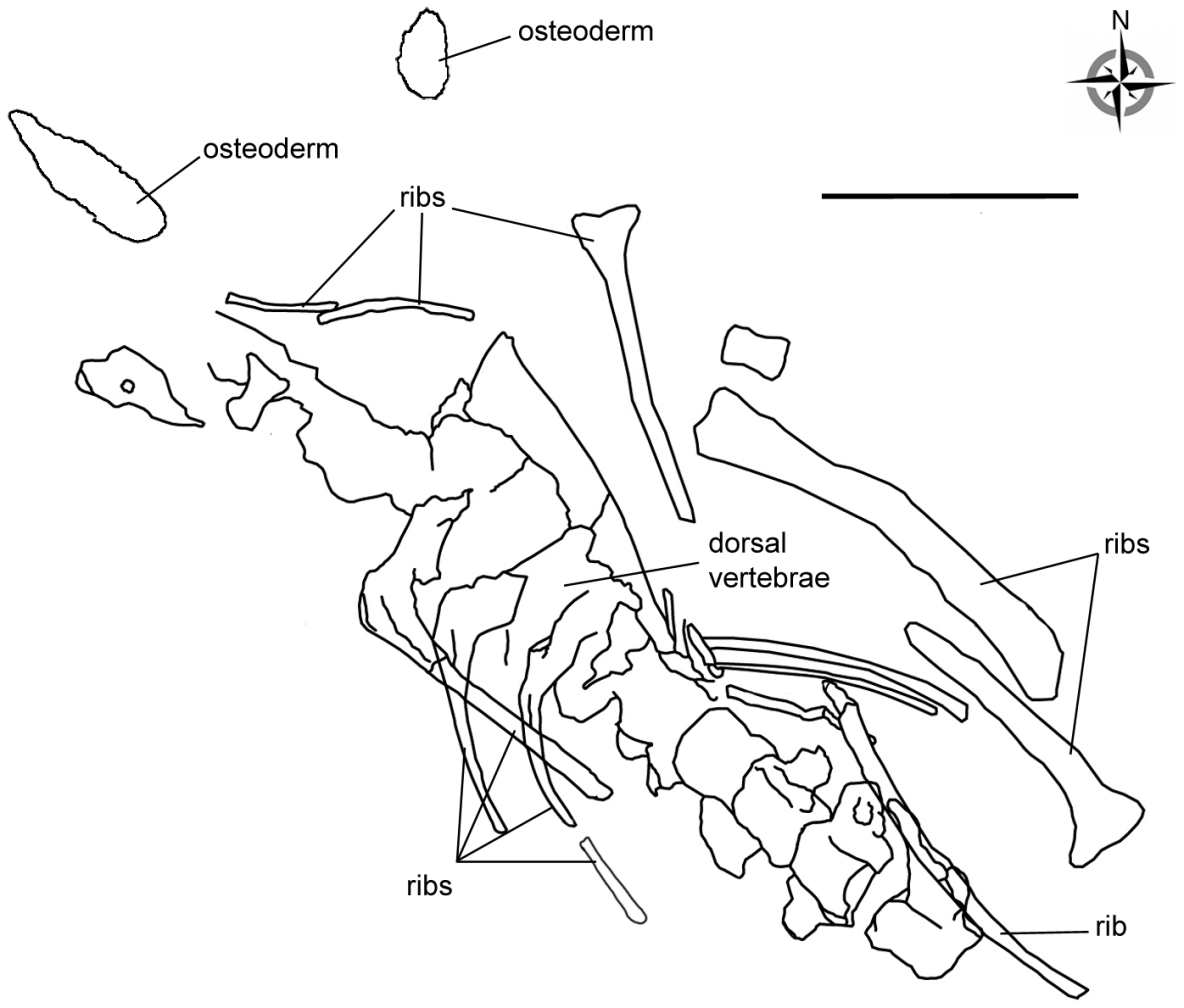
Dig site quarry map from the titanosaur HUE-EC-11. The main skeletal elements are highlighted and identified, as well as the osteoderms. Scale = 1 m.

The bulb and root osteoderms display extreme morphologies, yet continuum exists between those extremes (Figs. 6–7). Such a morphological cline is likely to represent the variability of one individual, as evidenced by two bulb and root osteoderms (HUE-00913 and HUE-00950) found associated with the same titanosaur individual at Lo Hueco (Fig. 10). These osteoderms were found a few meters away from each other and near the dorsal vertebrae of a single, isolated and partially articulated titanosaur specimen (HUE-EC-11), with the same orientation as the rest of long bones and with few elements of other taxa in the same bed. We consider, given the evidence, that HUE-00913 and HUE-00950 belonged to the same individual.

We interpret the morphological disparity from the EFA analysis as cline of intra-individual variability because i) one of the osteoderms associated with HUE-EC-11 is twice the size of its counterpart (Fig. 10); ii) both are located at different extremes of the morphological cline (Fig. 7); and iii) both belong to the same individual. This intra-individual variability would be similar to that displayed by the Stegosauria, where the size and shape of the dermal plates varies greatly among a single individual. How that intra-individual variation might have been arranged in titanosaurs, however, cannot be assessed from current evidence. Nevertheless, considering that locality data supports that titanosaurs would be lightly armored [2], a craniocaudal cline in two paramedian rows, as seen in stegosaurs, would be the most conservative conjecture. Alternative arrangements of more than two paramedian rows, each with its own craniocaudal cline could also be possible, but would require more osteoderms per individual. Nevertheless, osteoderms with such elongated roots have only been found at Lo Hueco thus far. It is possible that they may be a shared feature of Iberian forms, and thus other titanosaurs might have had slightly different arrangements.

## Supporting Information

Table S1 **Cartesian coordinates obtained for the osteoderm outlines employed in the elliptic Fourier analysis.**
(XLSX)

Table S2 **Institutional abbreviations of the osteoderms studied.**
(XLSX)
